# Prevalence of type 2 inflammation in patients with chronic rhinosinusitis with nasal polyps in Saudi Arabia

**DOI:** 10.3389/fsurg.2024.1421140

**Published:** 2024-07-10

**Authors:** Ahmad Aldajani, Ahmad Alroqi, Rana Alramyan, Nujud Alhejin, Mohammed Alswayyed, Waleed A. Alrajban, Saud Alromaih, Mohammad O. Aloulah, Abdulaziz S. Alrasheed, Surayie Aldousary, Saad Alsaleh

**Affiliations:** ^1^ Department of Otorhinolaryngology Head & Neck Surgery, College of Medicine, University of Jeddah, Jeddah, Saudi Arabia; ^2^ Department of Otolaryngology Head & Neck Surgery, College of Medicine, King Saud University, Riyadh, Saudi Arabia; ^3^ Department of Otorhinolaryngology Head & Neck Surgery, Security Forces Hospital, Riyadh, Saudi Arabia; ^4^ Department of Otorhinolaryngology Head & Neck Surgery, College of Medicine, King Salman Hospital, Riyadh, Saudi Arabia; ^5^ Department of Pathology and Laboratory Medicine, Collage of Medicine, King Saud University, Riyadh, Saudi Arabia

**Keywords:** chronic rhinosinusitis, nasal polyps, type 2 inflammation, eosinophils, cytokines, IgE, endoscopic sinus surgery, prevalence

## Abstract

**Background:**

Chronic Rhinosinusitis (CRS) is a common condition causing a significant worldwide burden, affecting 5%–12% of the general population. CRS is classified into type 2 and non-type 2 disease based on endotype dominance. Type 2 inflammation is distinguished by the presence of IL-4, IL-5, and IL-13 cytokines, along with eosinophil and mast cell activation and recruitment. Evidence of type 2 inflammation is ascertained by tissue eosinophil count >10/high-power field (HPF) or serum eosinophil >250 cells/mcL or total immunoglobulin E (IgE) > 100 IU/ml.

**Objectives:**

To investigate the prevalence and characteristics of type 2 inflammation in patients who presented with nasal polyps and underwent Endoscopic Sinus Surgery (ESS) in Saudi Arabia.

**Design:**

A retrospective cross-sectional Study.

**Methods:**

This study was conducted among patients who presented with nasal polyps and underwent ESS at King Saud University Medical City (KSUMC) from 2015 to 2020. Patients with nasal/sinus diseases other than Chronic Rhinosinusitis with Nasal Polyps (CRSwNP) were excluded. Demographic data, olfaction status, and co-morbidities were collected, and radiological images were evaluated. Type 2-CRS was determined by meeting at least one of three predictor criteria (blood eosinophils ≥250 cells/mcL, tissue eosinophils ≥10/HPF, or total IgE levels ≥100 IU/ml). Blood parameters and histopathologic analysis were obtained for each patient.

**Results:**

Of the 381 patients included in the study, the prevalence of type 2-CRS, based on the EPOS2020 criteria, was 99.7% in our population. Among these patients, 47.5% had hyposmia, 38.8% had anosmia, and 13.6% had normal olfaction. The most prevalent co-morbidity was allergic rhinitis, followed by bronchial asthma.

**Conclusion:**

This study aimed to determine the prevalence of type 2 inflammation among patients Diagnosed with CRSwNP and underwent ESS in Saudi Arabia. The results showed a prevalence of 99.7%, indicating that almost all recorded patients with CRSwNP in our population had type 2 inflammation.

## Introduction

Rhinosinusitis is a prevalent condition that imposes a substantial global burden in terms of healthcare utilization and productivity reduction. Chronic rhinosinusitis (CRS) causes more significant impairment in quality of life than acute rhinosinusitis, affecting 5%–12% of the general population. Furthermore, it is linked to bronchial asthma, with a prevalence of 25% in patients with chronic rhinosinusitis, compared to 5% in the general population (1). Rhinosinusitis is marked by the presence of symptomatic inflammation in the nasal cavity and paranasal sinuses. CRS in adults is characterized by the presence of two or more symptoms, such as nasal obstruction, nasal discharge, facial pressure, and decreased sense of smell, with nasal obstruction or nasal discharge being one of the symptoms for a duration of more than 12 weeks (2). The diagnosis of CRS can be supported by the utilization of nasal endoscopy and computed tomography (CT) scanning, with either one of them serving as a prerequisite for diagnosis. However, compared to clinical symptoms, endoscopic examinations, and imaging techniques, biomarkers are more objective, quantitative, and indicative of the underlying pathophysiology (3).

Prior to 2020, the differentiation of CRS subtypes was primarily based on clinical observations (4), emphasizing phenotypes, which refers to observable differences without a direct connection to the disease process (4), rather than focusing on the underlying histopathology or serum markers. The European Position Paper on Rhinosinusitis and Nasal Polyps 2020 (EPOS2020) group has opted to shift towards the endotype of CRS, which refers to distinct disease entities that may occur in clusters of phenotypes, but each defined by a specific biological mechanism (4). They chose to categorize CRS as primary and secondary, and further divide each into localized (unilateral) or diffuse (bilateral) based on the anatomical distribution. Most notably, both groups can be further classified into type 2 or non-type 2 disease based on endotype dominance. Primary localized CRS is then subdivided into two phenotypes: allergic fungal rhinosinusitis (AFRS) or isolated sinusitis. For diffuse CRS, the predominant clinical phenotypes are Eosinophilic Chronic Rhinosinusitis (eCRS) and Non-Eosinophilic Chronic Rhinosinusitis (non-eCRS), determined by the histologic quantification of eosinophil numbers per high-power field (HPF), which the EPOS panel agreed should be 10/HPF (at 400 × magnification) or higher. As for secondary CRS, the classification is again divided into localized or diffuse and then considered according to four categories depending on endotype dominance: local pathology, mechanical factors, inflammatory factors, or immunological factors (1).

As mentioned earlier, the current focus is on the resulting inflammation that occurs in the sinus tissue, rather than analyzing the complex and unknown factors that cause CRS in individual patients. The attention is shifting towards identifying the molecular pathways or endotypes that become activated. With the advent of modern biological techniques, it has become possible to adopt a more mechanistic approach in the diagnosis and treatment of CRS by aiding the comprehension of critical cellular processes and immunological responses against pathogens across mucosal barriers (5). When the barrier is breached, a self-limited immune-defensive response is generated, which is characterized by a cellular and cytokine repertoire that targets one of the three classes of pathogens: type 1 immune responses target viruses; type 2 responses target parasites; and type 3 responses target extracellular bacteria and fungi. All of these responses typically resolve with the elimination of the pathogen and the restoration of barrier integrity. However, in CRS, the barrier penetration results in a chronic inflammatory response that fails to resolve but still usually involves the type 1, 2, or 3 pathways alone, or in combinations (1).

Type 2 inflammation is characterized by cytokines such as IL-4, IL-5, and IL-13, along with the activation and recruitment of eosinophils and mast cells. The presence of type 2 inflammation is identified by a tissue eosinophil count of >10/HPF, serum eosinophil count of >250 cells/mcL, or total immunoglobulin E (IgE) level of >100 IU/ml. Recent research has shown that patients with a pure or mixed type 2 endotype tend to be more resistant to current therapies, with a higher recurrence rate than those with pure type 1 or 3 endotypes (1).

Multiple studies conducted in Western regions have shown a higher incidence of type 2 CRS in patients with nasal polyps, with eosinophils being the dominant inflammatory cell (6). In contrast, studies conducted in Eastern Asia have shown a higher incidence of non-type 2 CRS, with neutrophils being the dominant inflammatory cell in nasal polyps (7–9). The current study aims to focus on the type 2 endotype and determine its prevalence and characteristics among patients with Chronic Rhinosinusitis with Nasal Polyps (CRSwNP) who underwent ESS in Saudi Arabia.

## Methodology

### Study design

A retrospective cross-sectional study was conducted among patients who presented with nasal polyps and underwent Endoscopic Sinus Surgery (ESS) at King Saud University Medical City in Riyadh, Saudi Arabia, between 2015 and 2020. Patients’ information was collected confidentially using the hospital's electronic system for integrated health information (eSiHi).

### Study population

The study included a cohort of 552 patients who presented with nasal polyps and underwent ESS between 2015 and 2020. Of these, 171 patients were excluded from the analysis due to the presence of nasal/sinus conditions other than CRSwNP or the absence of intraoperative sinonasal tissue biopsy. As a result, the final sample consisted of 381 patients (mean age: 41 ± 15) who were diagnosed with CRSwNP according to EPOS2020 (5) and underwent ESS as part of their disease management.

The single population proportion formula was used to estimate the sample size. A confidence level of 95% with a margin of error of 5% was employed to determine the needed sample size. A previous study conducted in Saudi Arabia reported the frequency of histopathological changes in nasal polyps among patients with Chronic Rhinosinusitis (10). To achieve a 95% power and a type-I error rate of 0.05, the required sample size for the study was estimated to be 303 patients, which was surpassed in our study. The calculated sample size resulted in a sufficient number of participants with a range of characteristics, generating a sample that was representative of the population. Moreover, as KSUMC is considered one of the largest subspecialised centres in the Middle East, receiving and treating patients from all over the region, and to the best of our knowledge, no previous study has investigated the prevalence and characteristics of type 2 inflammation in the Middle East region, we considered our population to be representative of the Middle Eastern region.

### Study outcomes

#### General health information

Demographic characteristics, including age, gender, smoking status, and medical history, were recorded. Bronchial asthma and olfaction status were also collected. Determination of asthma was based on the selection of self-reported physician-diagnosed asthma patients from medical records. Olfaction status was defined subjectively by the degree of function as normal, hyposmia or anosmia. Allergic rhinitis symptoms were also subjectively recorded. Patients' comorbidities, such as hypertension (HTN), diabetes mellitus (DM), dyslipidemia (DLD), and chronic obstructive pulmonary disease (COPD), were included. The presence of polyps was assessed via nasal endoscopy during the first clinic visit. Radiological images were evaluated using the Lund & Mackay grading system, in addition to olfactory cleft opacification grading and the presence of hyperdensities.

#### Determination of type 2 inflammation in patients with nasal polyps

Sinonasal polyps were obtained in specimens intraoperatively and preserved in formalin. These were then processed with standard hematoxylin and eosin staining. Additionally, an expert pathologist, who was blinded to the clinical data, re-examined the previously collected specimens in a prospective manner. Type 2-CRS was defined in accordance with EPOS2020 guidelines, with a patient meeting at least one of three predictors' criteria (blood eosinophils ≥ 250 cells/mcL, tissue eosinophils ≥ 10/HPF, or total IgE levels ≥ 100 IU/ml). Moreover, type 2 tissue eosinophils were classified as subgroups from mild (0–50), moderate (51–100) to severe (>100).

Each sinonasal specimen underwent analysis for various parameters including tissue eosinophil count (quantification of eosinophil number per HPF at 400 × magnification), neutrophilic infiltrate (categorized as absent, diffuse, or focal), inflammatory cell predominance (classified as neutrophilic, lymphohistiocytic, eosinophilic, lymphoplasmacytic, or lymphocytic), sub-epithelial oedema (graded as absent, mild, moderate, or severe), hyperplastic/papillary change (noted as absent or present), mucosal ulceration (noted as absent or present), squamous metaplasia (noted as absent or present), fibrosis (noted as absent or present), Graccot's Methenamine Silver (GMS) stain (noted as absent, present, or not performed), fungal elements (noted as absent or present), Charcot-Leyden crystals (noted as absent or present), and eosinophil aggregates (noted as absent or present) (4).

A blood sample was taken preoperatively and assessed for various markers, including serum eosinophil ratio (%), total eosinophil count (cells/mcL), and total IgE (IU/ml). The eosinophil ratio was calculated by dividing the eosinophil count by the white blood cell count and expressed as a percentage. All parameters were assessed in a blinded manner.

A bacterial and fungal culture swab was obtained intraoperatively from the sinonasal cavity for all patients. The results were categorised as positive or negative based on the presence or absence of organism growth. Additionally, the names of the cultured organisms were recorded and reported.

### Statistical analysis

The Statistical Package for Social Sciences (SPSS) version 23.0 was utilized. The data set was cleaned and coded for inconsistencies. Both descriptive and analytical analyses were performed. The data were tested for normality using the Kolmogorov-Smirnov test, as well as skewness and kurtosis, and represented graphically. Mean and standard deviation were used to express parametric results, while frequencies were used for categorical data.

### Research ethics

This study received ethical approval from the Institutional Review Board Committee at the College of Medicine, King Saud University (Approval No. E-21-5821). Identification data were not used in this study, and we ensured that all personal information of the patients was kept confidential and protected. Written informed consent was obtained from all subjects included in the study.

## Results

### Prevalence and characteristics of type 2 inflammation based on EPOS2020 criteria

Out of the 381 patients with CRSwNP, 58.8% were male and 41.2% were female, with a mean age of 41 ± 15 years. More than half of the patients (56.2%) had no comorbidities, and the majority were non-smokers. Culture results were negative in 42% and positive in 31% of cases. Olfaction status was recorded, with 47.5% of the study population being hyposmic, 38.8% being anosmic, and only 13.6% having normal olfaction. Remarkably, the utilization of EPOS2020 criteria for type 2 inflammation in our population revealed that 99.7% exhibited type 2 inflammation, while only 0.3% displayed non-type 2 inflammation. This indicates that nearly all the recorded patients with CRSwNP who underwent ESS had type 2 inflammation. (Table 1)

**Table 1:** Prevalence of type 2 and socio-demographic characteristics of patients with CRSwNP (*N* = 381).

#	Sample Characteristics	*N* (%)	M ± SD	
1	Gender			
	Male	224 (58.8)		
	Female	157 (41.2)		
2	Age		41 ± 15	
3	Culture			
	Negative	163 (42.8)		
	Positive	121 (31.8)		
	Not performed	97 (25.5)		
4	Co-morbidity			
	Medically free	214 (56.2)		
	Medically condition	167 (43.8)		
5	Smoking			
	No	350 (91.9)		
	Yes	31 (8.1)		
6	Olfaction			
	Hyposmia	181 (47.5)		
	Anosmia	148 (38.8)		
	Normal	52 (13.6)		
7	Endotype based on EPOS2020			
	Type2	380 (99.7)		
	Non-type2	1 (0.3)		
8	Prevalence of co-morbidities			
	Bronchial asthma	126 (33.1)		
	DM	51 (13.4)		
	HTN	48 (12.6)		
	DLD	31 (8.1)		
	COPD	1 (0.3)		
	Allergic rhinitis	211 (55.4)		

Co-morbidities were illustrated among patients with CRSwNP, including past or current health issues. Allergic rhinitis was more prevalent (55.4%) than other medical co-morbidities, followed by bronchial asthma (33.1%). The prevalence of chronic diseases was 13.4%, 12.6% and 8.1% for diabetes mellitus, hypertension, and dyslipidaemia, respectively. (Table 1)

We present the laboratory measurements of blood, radiological, and histopathological tests conducted on patients with CRSwNP. The mean tissue eosinophil count was 68 ± 36/HPF, and the total eosinophil count and total IgE were also high, with values of 374.2 ± 347.7 cells/mcL and 519.1 ± 464.7 IU/ml, respectively. The mean Lund & Mackay score was 17 ± 6, and the total olfactory cleft opacification had a score of 5 ± 3 out of a total of 8. Hyperdensities were observed in approximately half of the patients (51.1%). Neutrophilic infiltrate was diffuse in around 60% of cases and focal in 15%. Lymphohistiocytic inflammatory cell predominance was observed in 37.8% of cases, while eosinophilic and lymphoplasmocytic infiltrates were present in around one-third of cases each (28.1% and 29.1%, respectively). Fibrosis was detected in almost half of the patients (49.1%), while hyperplastic changes were seen in 42.3%, and mucosal ulceration was observed in one-third of cases (32.5%). Furthermore, a low prevalence of fungal elements (11%), Charcot-Leyden crystals (18.9%), and eosinophil aggregates (17.6%) was noted compared to other results. (Table 2)

**Table 2:** Laboratory, radiological and histopathology measurements among patients with CRSwNP.

#	Characteristics	M ± SD
1	Serum biomarkers	
	Serum eosinophil (%)	8.83 ± 22.49
	Total eosinophil count (cells/mcL)	374.2 ± 347.7
	Total IgE (IU/ml)	519.1 ± 464.7
2	CT test (radiology)	
	Lund & mackay score 0–24	17 ± 6
	Anterior olfactory cleft opacification 0–4	3 ± 2
	Posterior olfactory cleft opacification 0–4	3 ± 1
	Total olfactory cleft opacification 0–8	5 ± 3
3	Histopathology test	
	Tissue eosinophil count (number/HPF)	68 ± 36
#	Histopathological Characteristics	N (%)
1	Neutrophilic infiltrate	
	Absent	96 (25.2)
	Diffuse	228 (59.8)
	Focal	57 (15.0)
2	Inflammatory cell predominance	
	Neutrophilic	10 (2.6)
	Lymphohistiocytic	144 (37.8)
	Eosinophilic	107 (28.1)
	Lymphoplasmocytic	111 (29.1)
	Lymphocytic	9 (2.4)
3	Sub-epithelial oedema	
	Absent	8 (2.1)
	Mild	85 (22.3)
	Moderate	180 (47.2)
	Severe	108 (28.3)
4	Hyperplastic/Papillary change	
	Absent	220 (57.7)
	Present	161 (42.3)
5	Mucosal ulceration	
	Absent	257 (67.5)
	Present	124 (32.5)
6	Squamous metaplasia	
	Absent	271 (71.1)
	Present	110 (28.9)
7	Fibrosis	
	Absent	194 (50.9)
	Present	187 (49.1)
8	Graccot's Methenamine Silver (GMS) Stain	
	Negative	104 (27.3)
	Positive	29 (7.6)
	Not performed	248 (65.1)
9	Fungal elements	
	Absent	339 (89.0)
	Present	42 (11.0)
10	Charcot-Leyden crystals	
	Absent	309 (81.1)
	Present	72 (18.9)
11	Eosinophil aggregates	
	Absent	314 (82.4)
	Present	67 (17.6)

We have conducted an analysis of the culture swab results from patients with CRSwNP. Among the samples, Staphylococcus aureus was the most prevalent organism, accounting for 9.2%. It was followed by Aspergillus flavus (5.6%), Klebsiella (4.9%), and Bipolaris (3.5%). Approximately 3% of the samples tested positive for methicillin-resistant Staphylococcus aureus (MRSA), 3% tested positive for Pseudomonas aeruginosa, and 2.1% tested positive for Escherichia coli (E. coli). (Table 3)

**Table 3:** Prevalence of cultured organisms among patients with CRSwNP (*N* = 284).

#	Characteristics	*N*	%
	Bacterial		
1	Staph Aureus	26	9.20%
2	Klebsiella (oxytoca or pneumonia)	14	4.90%
3	Citrobacter koseri	8	2.80%
4	MRSA	8	2.80%
5	Pseudomonas Aeruginosa	8	2.80%
6	E.Coli	6	2.10%
7	Enterobacter Aerogenes	5	1.80%
8	Serratia macescens	3	1.10%
9	Hemophilus influenza	2	0.70%
10	Enterobacter Cloacae	2	0.70%
11	Proteus mirabilis	1	0.40%
12	Sphingomonas Paucimbilis	1	0.40%
13	Enterococcus Faecalis	1	0.40%
14	Streptococcus dysgalactiae	1	0.40%
15	Streptococcus pyogenes group A	1	0.40%
	Fungal		
1	Aspergillus flavus	16	5.60%
2	Bipolaris fungal	10	3.50%
3	Curvularia fungi	4	1.40%
4	Aspergillus fumigatus	2	0.70%
5	Aspergillus niger	2	0.70%
6	Unspecified fungal	2	0.70%
7	Asperigillus terreus	1	0.40%
8	Fungal Trichoderma	1	0.40%
9	Hyaline fungus	1	0.40%

### Prevalence of type 2 inflammation based on tissue eosinophil count, total serum eosinophil count and total IgE

The endotyping of CRS based on tissue eosinophil count showed that 93.4% of our patients had type 2 disease, while only 6.6% had non-type 2 disease. Among the type 2 patients, the majority belonged to the severe subgroup (41.2%), followed by the mild and moderate subgroups, which accounted for 31% and 21.3% respectively. (Table 4)

**Table 4:** Prevalence of type 2 inflammation based on tissue eosinophils, serum eosinophils and total IgE.

Based on tissue eosinophil count	*N*	%
Type2	356	93.4
Non-type2	25	6.6
Tissue eosinophil subgroups		
Mild (0–50)	118	31
Moderate (51–100)	81	21.3
Severe (>100)	157	41.2
Based on total eosinophil count		
Type 2	235	61.7
Non-Type2	146	38.3
Based on total IgE		
Type 2	341	89.5
Non-Type2	40	10.5

When the endotype of CRSwNP was identified based on the total serum eosinophil count, the majority of cases were found to be type 2, with a prevalence of 61.7%, while non-type 2 cases accounted for 38.3%. In contrast, based on IgE results, more than three-quarters of patients (89.5%) were classified as type 2, while only 10.5% were classified as non-type 2. (Table 4)

In this study, we examined the type 2 tissue eosinophil subgroups and their corresponding patient characteristics. The severity of type 2 eosinophils was classified as severe (>100), moderate (51–100), and mild (0–50). The demographic factors, comorbidities, olfactory status, laboratory findings, and CT findings were measured, and no significant differences were observed among the three classes of tissue eosinophil subgroups.

## Discussion

Following the new EPOS classification published in 2020 (1), our study found that 99.7% of patients in our population had type 2 inflammation, and only 0.3% representing one patient had non-type 2 inflammation, indicating that nearly all recorded patients with CRSwNP had type 2 inflammation. Similarly, Stevens et al. reported that among CRSwNP patients, the overall frequency of any type 1, type 2, or type 3 inflammation was 17%, 87%, and 18%, respectively, of which 0.7%, 62%, or 2.2% had only type 1, type 2, or type 3 single inflammation (11). In American and European patient cohorts, CRSwNP strongly leans towards a type 2 response (12–14). However, this relationship is not observed in Asian populations. Instead, Asian CRSwNP populations, with most data coming from China, tend towards neutrophilic inflammation (15, 16). Moreover, the non-eosinophilic type has been considered the most common subgroup of CRSwNP in Japan for the past 30 years (7).

There is a general consensus in the literature that CRSwNP is often associated with comorbid asthma (17–26). In this study, our data reported an association with bronchial asthma (33.1%), but allergic rhinitis was more prevalent (55.4%). Considering comorbidities can provide a useful tool for understanding the disease, given the shared inflammatory mechanisms between comorbidities and CRS, and the potential immunomodulation of CRS by other inflammatory processes.

As reported in the literature, we found that 47.5% of the patients in our study were hyposmic, 38.8% were anosmic, and only a minority of patients (13.6%) had a normal olfaction. Similarly, Stevens et al. reported significantly higher complaints of smell/taste loss in eosinophilic CRS, with 78% of patients having CRSwNP (11). Other studies have also suggested that type 2 inflammation promotes smell loss in CRS (27, 28). However, loss of smell is well known to be associated with the presence of nasal polyps (1), and therefore, it is not clear whether the type 2 inflammation was merely a marker of nasal polyps or whether the type 2 inflammation was more directly associated with smell disturbance.

Ho et al. conducted a retrospective study on 345 patients, of whom 206 (59.7%) had eosinophilic CRS. They found that eosinophils were significantly higher in the eCRS population compared to the non-eCRS group (*P* < .01) (29). Similar findings were observed in our study, with a mean total eosinophil count of 374.2 ± 347.7 cells/mcL in patients with type 2 inflammation.

Given the high costs, both financial and in terms of implementation, associated with molecular diagnosis and biological treatment, it is of immense importance to identify suitable biomarkers and develop endotype-driven therapies for the management of CRSwNP (1, 30). Biologics that specifically target type 2 inflammation have become the primary focus of ongoing clinical trials for CRSwNP. These trials have shown a progressive increase in the development of biologic therapies that target the pivotal molecules involved in type 2 inflammation. Based on the existing research, biologics, in general, have demonstrated the potential to alleviate the characteristic symptoms of CRSwNP, including nasal obstruction, rhinorrhea, and impaired olfactory function, thereby significantly enhancing patients' overall quality of life (31–33). Biologics have continued to cause a paradigm shift in the management of the disease since their introduction for clinical use.

As previously mentioned, calculating the prevalence of type 2 inflammation in patients with CRSwNP using the EPOS2020 criteria resulted in a prevalence of 99.7%. In comparison, determining the prevalence based on tissue eosinophil count, total eosinophil count, or total IgE separately showed a prevalence of 93.4%, 61.7%, and 89%, respectively. These findings underscore the importance of adhering to the EPOS2020 criteria for determining the type 2 endotype in patients with CRSwNP.

### Strengths and limitations

The major strength of this study is that it is the first to address the prevalence of type 2 inflammation in patients with CRSwNP who underwent ESS in the Middle East region. Furthermore, the study concluded that nearly all nasal polyp patients in our population exhibit type 2 inflammation. However, there are several limitations that should be acknowledged. Firstly, it was conducted at a single center, which may limit the generalizability of the findings. Secondly, the study sample only consisted of patients diagnosed with CRSwNP and did not include patients with CRS without nasal polyps, potentially impacting the overall representation of CRS cases. Additionally, the study focused exclusively on type 2 and non-type 2 endotypes without specifying whether the non-type 2 endotypes were classified as type 1 or type 3. Moreover, important information regarding medical treatments prior to surgery, such as the use of oral corticosteroids (OCS), may have not been reported, which could have influenced the study outcomes. Nevertheless, nearly all patients were endotyped to exhibit type 2 inflammation, and research has indicated that the administration of pre-operative OCS could potentially reduce the presence of tissue eosinophils (34). Furthermore, another limitation is the absence of formal endotyping utilizing cytokine markers such as IL-4, IL-13, ECP, etc., which were not accessible during the study. Additionally, Olfaction status and allergic rhinitis in our study were based on self-reported physician diagnoses and not on objective tests, as olfaction tests were not available at the time of the study, and skin prick tests were not documented for all patients in our database. Lastly, the study excluded patients with nasal polyps who did not undergo sinus surgery, as all of our CRSwNP patients required ESS as part of their treatment, limiting the inclusion of a broader population.

## Conclusion

This study aimed to determine the prevalence and characteristics of type 2 inflammation among patients diagnosed with CRSwNP who underwent ESS in Saudi Arabia, based on the EPOS2020 criteria. Our study population revealed that 99.7% of patients had type 2 inflammation, and only one patient (0.3%) had non-type 2 inflammation, indicating that nearly all recorded patients with CRSwNP in our population had type 2 inflammation. As a priority, health policy should encourage more studies on the prevalence and characteristics of type 2 inflammation in CRSwNP patients and increase awareness of type 2 inflammation as it relates to nasal polyps in the Middle East.

## Data Availability

The raw data supporting the conclusions of this article will be made available by the authors, without undue reservation.
